# Global Distribution and Evolution of *Mycobacterium bovis* Lineages

**DOI:** 10.3389/fmicb.2020.00843

**Published:** 2020-05-07

**Authors:** Cristina Kraemer Zimpel, José Salvatore L. Patané, Aureliano Coelho Proença Guedes, Robson F. de Souza, Taiana T. Silva-Pereira, Naila C. Soler Camargo, Antônio F. de Souza Filho, Cássia Y. Ikuta, José Soares Ferreira Neto, João Carlos Setubal, Marcos Bryan Heinemann, Ana Marcia Sa Guimaraes

**Affiliations:** ^1^Laboratory of Applied Research in Mycobacteria, Department of Microbiology, Institute of Biomedical Sciences, University of São Paulo, São Paulo, Brazil; ^2^Department of Preventive Veterinary Medicine and Animal Health, School of Veterinary Medicine and Animal Sciences, University of São Paulo, São Paulo, Brazil; ^3^Department of Biochemistry, Institute of Chemistry, University of São Paulo, São Paulo, Brazil; ^4^Laboratory of Cellular Cycle, Butantan Institute, São Paulo, Brazil; ^5^Laboratory of Protein Structure and Evolution, Department of Microbiology, Institute of Biomedical Sciences, University of São Paulo, São Paulo, Brazil; ^6^Biocomplexity Institute of Virginia Tech, Blacksburg, VA, United States

**Keywords:** *Mycobacterium bovis*, genomic, evolution, lineage, bovine tuberculosis (bTB)

## Abstract

*Mycobacterium bovis* is the main causative agent of zoonotic tuberculosis in humans and frequently devastates livestock and wildlife worldwide. Previous studies suggested the existence of genetic groups of *M. bovis* strains based on limited DNA markers (a.k.a. clonal complexes), and the evolution and ecology of this pathogen has been only marginally explored at the global level. We have screened over 2,600 publicly available *M. bovis* genomes and newly sequenced four wildlife *M. bovis* strains, gathering 1,969 genomes from 23 countries and at least 24 host species, including humans, to complete a phylogenomic analyses. We propose the existence of four distinct global lineages of *M. bovis* (Lb1, Lb2, Lb3, and Lb4) underlying the current disease distribution. These lineages are not fully represented by clonal complexes and are dispersed based on geographic location rather than host species. Our data divergence analysis agreed with previous studies reporting independent archeological data of ancient *M. bovis* (South Siberian infected skeletons at ∼2,000 years before present) and indicates that extant *M. bovis* originated between 715 and 3,556 years BP, with later emergence in the New World and Oceania, likely influenced by trades among countries.

## Introduction

Tuberculosis (TB) is the leading infectious killer in the world and approximately 10 million new cases are reported annually. In 2017, 1.6 million people died of TB and over 95% of these deaths occurred in low and middle-income countries (World Health Organization [WHO], 2018). The disease is strongly linked to poverty, with its prevalence following a socioeconomic gradient within and among countries (Lönnroth et al., 2009). In addition, there is a significant, and often neglected, contributor to the global disease burden, which is the zoonotic transmission of bovine TB to humans (Olea-Popelka et al., 2017). The WHO (World Health Organization) estimated that 142,000 new cases and 12,500 deaths occurred due to zoonotic TB in 2017 (World Health Organization [WHO], 2018), numbers that are likely underestimated due to lack of routine surveillance data from most countries (Olea-Popelka et al., 2017). People with zoonotic TB face arduous challenges; most strains of the etiologic agent are resistant to pyrazinamide (Konno et al., 1967; Scorpio and Zhang, 1996; Loiseau et al., 2019), one of the first-line drugs used in TB treatment, and a possible association with extra-pulmonary disease (Dürr et al., 2013) often delays diagnostics and treatment initiation (World Health Organization [WHO] et al., 2017). In addition, bovine TB results in severe economic losses for livestock producers worldwide, respecting no borders and repeatedly affecting animal conservation efforts due to the establishment of wildlife reservoirs or spillover events from cattle to associated animal populations (Ayele et al., 2004; De Kantor and Ritacco, 2006; Godfray et al., 2013; Miller and Sweeney, 2013; Palmer, 2013; Nugent et al., 2015a, b). In order to eradicate TB by 2,030 as part of the United Nations (UN) Sustainable Development Goals, it is imperative that future prevention and control strategies focus on all forms of TB in humans, including its interface with animals.

Human and animal TB are caused by members of the *Mycobacterium tuberculosis* Complex (MTBC). The MTBC is a clonal bacterial group composed of 12 species or ecotypes with variable virulence and host tropism (Galagan, 2014). *Mycobacterium tuberculosis stricto sensu* is the main responsible for the TB numbers and is adapted to human hosts (Brites and Gagneux, 2015; Malone and Gordon, 2017). On the other hand, *Mycobacterium bovis*, the causative agent of bovine TB, has a broader host range and is able to infect and cause disease in multiple host species, including humans, with variable populational persistence (Malone and Gordon, 2017). MTBC members have clonally evolved from a common ancestor with the tuberculous bacteria *Mycobacterium canettii* (Supply et al., 2013), and alignable regions of MTBC genomes are over 99.95% identical, with horizontal gene transfer and large recombination events considered absent (Hirsh et al., 2004; Gagneux and Small, 2007; Galagan, 2014). These pathogens have solely evolved through single nucleotide polymorphisms (SNPs), indels, deletions of up to 26 Kb, duplication of few paralogous genes families, and insertion sequences (IS), which translated into a phenotypic array of host tropism and virulence variations (Brosch et al., 2002; Gagneux and Small, 2007; Lazzarini et al., 2007; Galagan, 2014; Brites et al., 2018).

Using whole-genome, SNP-based phylogenetic analyses, human-adapted MTBC have been classified into 7 lineages, with *M. tuberculosis* accounting for L1 to L4 and L7, and *Mycobacterium africanum* comprising L5 and L6 (Coscolla and Gagneux, 2014). Each human-adapted MTBC lineage is associated with specific global geographical locations, and lineage-associated variations in virulence, transmission capacity and in the propensity to acquire drug resistance have been reported (de Jong et al., 2010; Portevin et al., 2011, 2014; Gagneux, 2012; Sarkar et al., 2012; Coscolla and Gagneux, 2014). Thus, regional prevalence of specific lineages or sub-lineages have consequences for the epidemiology of TB worldwide. A similar attempt to classify *M. bovis* into different genetic groups was made prior to the large-scale availability of whole-genome sequences and started with the identification of clonal complexes (CCs). Accordingly, four *M. bovis* CCs have been described (African 1 and 2, European 1 and 2), and these are determined based on specific deletions ranging from 806 to 14,094 bp (base pairs), SNPs and spoligotypes (Müller et al., 2009; Berg et al., 2011; Smith et al., 2011; Rodriguez-Campos et al., 2012). As with *M. tuberculosis* lineages, *M. bovis* CCs appear to have distinct geographical distributions, with African 1 and 2 restricted to Africa, European 2 commonly found in the Iberian Peninsula, and European 1 distributed globally (Müller et al., 2009; Berg et al., 2011; Smith et al., 2011; Rodriguez-Campos et al., 2012). Although there are no studies specifically aimed at identifying differences in virulence patterns among *M. bovis* of different CCs, numerous articles report virulence variations among strains of *M. bovis* (Wedlock et al., 1999; Waters et al., 2006; Meikle et al., 2011; Wright et al., 2013; de la Fuente et al., 2015; Vargas-Romero et al., 2016), suggesting a possible link between bacterial genetic polymorphisms and disease development, as observed in *M. tuberculosis*.

Since the whole-genome sequence of the first *M. bovis* strain became available in 2003 (Garnier et al., 2003), increasing efforts have been made to sequence additional strains and use whole-genome information to tackle bovine and/or wildlife TB transmission within specific outbreaks or countries (Bruning-Fann et al., 2017; Sandoval-Azuara et al., 2017; Ghebremariam et al., 2018; Kohl et al., 2018; Lasserre et al., 2018; Orloski et al., 2018; Price-Carter et al., 2018; Razo et al., 2018). However, no studies to date have comprehensively analyzed *M. bovis* genomes at a global scale to provide insights into its populational structure and evolution based on whole-genome information. Few studies that have compared transboundary *M. bovis* strains analyzed bacterial isolates obtained from a reduced number of countries (*n* < 9) and included small sample sizes (Dippenaar et al., 2017; Patané et al., 2017; Zimpel et al., 2017a; Ghebremariam et al., 2018; Lasserre et al., 2018). Nevertheless, attained results suggest that *M. bovis* strains are likely to cluster based on geographical location (Dippenaar et al., 2017; Zimpel et al., 2017a; Lasserre et al., 2018). In our previous study, we have also shown that few *M. bovis* genomes do not carry any CC genetic marker (Zimpel et al., 2017a), a phenomenon that was recently observed in *M. bovis* isolates from one cattle herd in the United States and from slaughterhouse cattle in Eritrea (Ghebremariam et al., 2018; Orloski et al., 2018). These findings suggest that CCs are unlikely to represent the whole diversity of *M. bovis* strains, warranting further evaluation of *M. bovis* molecular lineages (Zimpel et al., 2017a; Lasserre et al., 2018). Therefore, the aims of this study were to perform a phylogenomic analysis to understand the populational structure of *M. bovis* worldwide and to provide dating estimates for the origin of this important pathogen.

We have screened over 2,600 publicly available *M. bovis* genomes and newly sequenced four wildlife *M. bovis* strains, gathering 1,969 *M. bovis* genomes from 23 countries and at least 24 different host species, including humans, to complete a phylogenomic analyses. Our phylogenetic reconstruction suggests the existence of at least four distinct lineages of *M. bovis* in the world. We also evaluated the evolutionary origin of *M. bovis* strains and lineages and correlated bacterial population dynamics with historical events to gain new insights into the widespread nature of bovine TB worldwide.

## Materials and Methods

### Genome Sequencing of Brazilian *M. bovis* Genomes

Four Brazilian *M. bovis* isolates obtained from one captive European bison (*Bison bonasus*) (Zimpel et al., 2017b), two captive llamas (*Llama glama*), and one captive capybara (*Hydrochoerus hydrochaeris*) (provided by the Laboratory of Bacterial Zoonosis of the College of Veterinary Medicine, University of São Paulo, Brazil) were reactivated in Stonebrink medium and a single colony was sub-cultured for DNA extraction using a previously described protocol (Zimpel et al., 2017a). DNA quality was evaluated using Nanodrop 2000c (Thermo Scientific, MA, United States) and Agilent 2100 High Sensitivity Chip DNA Bioanalyzer (Agilent Technologies, CA, United States). All procedures involving live tuberculous mycobacteria were performed in a Biosafety Level 3+ Laboratory (BSL-3+ Prof. Dr. Klaus Eberhard Stewien) located at the Department of Microbiology, Institute of Biomedical Sciences, University of São Paulo, Brazil.

Paired-end genomic libraries were constructed using TruSeq DNA PCR-free sample preparation kit (Illumina, CA, United States), and Illumina HiSeq2500 (Illumina v3 chemistry) was used to sequence the genomic library (100 bp). These procedures were performed at the Central Laboratory of High Performance Technologies in Life Sciences (LaCTAD), State University of Campinas (UNICAMP), São Paulo, Brazil. Illumina sequencing reads were deposited in the Sequence Read Archive (SRA) from the National Center for Biotechnology Information (NCBI) (accession numbers: SRR7693912, SRR7693877, SRR9850824, and SRR9850830).

### Selection of *M. bovis* Genomes

We searched for genomes identified as “*Mycobacterium bovis* not BCG” deposited in SRA, NCBI. At the time of this selection (September 2018), the designation “*Mycobacterium tuberculosis* variant *bovis”* had not yet been applied. Accordingly, there were approximately 2,600 sequence read sets of *M. bovis* genomes deposited in this database. Genomes from *M. bovis* were initially selected if they: (i) presented known geographic location based on SRA metadata or associated publications; and (ii) were virulent *M. bovis* strains, i.e., not named as *M. bovis* AN5, *M. bovis* Ravenel, and were not re-sequencing files of the reference genome used herein, *M. bovis* AF2122/97 (Supplementary Figure S1). We focused on *M. bovis* genomes with metadata regarding location because one of our goals was to address the global distribution of the pathogen at country/continent level. Host species information were retrieved from SRA metadata or associated publications.

We also selected 30 additional *M. bovis* genomes that were sequenced or identified after September 2018 by Brites et al. (2018). These isolates were from Germany (*n* = 7), Ghana (*n* = 5), Malawi (*n* = 3), Republic of Congo (*n* = 3), Russia (*n* = 2), Switzerland (*n* = 4), and United Kingdom (*n* = 6). The same inclusion criteria described above was followed in the selection of these genomes. In the end, a total of 2,202 virulent *M. bovis* genomes were initially selected using the above described methods and criteria (Supplementary Table S1 and Supplementary Figure S1).

### Sequencing Quality Criteria

FASTQ files of all 2,202 initially selected *M. bovis* genomes were downloaded from SRA, NCBI and trimmed using Trimmomatic (Sliding window: 5:20) (Bolger et al., 2014). To guarantee the selection of genomes with good sequence quality, the following quality criteria were considered after trimming: (i) coverage ≥ 15x, (ii) median read length of at least 70 bp, (ii) absence of low quality sequences and anomalous GC content as determined using FastQC, and (iv) mapping coverage against reference genome *M. bovis* AF2122/97 (NC_002945.4) greater than 95% as indicated by using Burrows-Wheeler Aligner (Li and Durbin, 2010). A customized script (Snps_pipeline; #check depth in RD positions; available in github – see below) was developed to confirm that all genomes were of *M. bovis* species and not BCG or other MTBC species by searching for the presence of RD1 and RD4 deletions, respectively (RD stands for “regions of difference,” Mostowy et al., 2002). Corresponding regions were evaluated by checking their sequencing coverage when mapping each read set against the reference genome of *M. tuberculosis* H37Rv using Burrows-Wheeler Aligner (Li and Durbin, 2010). The following positions, based on *M. tuberculosis* H37Rv reference genome, were used: RD1 (4,354,000 – 4,358,331) and RD4 (1,703,743 – 1,705,033). A region was considered deleted when average coverage was below four for RD1 or 15 for RD4, as described previously (Faksri et al., 2016).

### Mapping and Variant Calling of Reads

Each quality-approved read set was mapped against the reference genome *M. bovis* AF2122/97 (NC_002945.4) using Burrows-Wheeler Aligner (Li and Durbin, 2010). Duplicated reads were removed using Picard v2.18.23^1^. SNPs were called using Samtools v1.9 mpileup (Li, 2011), and VarScan v2.4.3 mpileup2cns (Koboldt et al., 2012), using parameters of read depth of 7, minimum mapping quality and minimum base quality of 20. Generated vcf files were annotated using snpEff (Cingolani et al., 2012) based on the same reference genome, and manipulated using awk programming language to remove SNPs located in PE/PPE, transposase, integrase, maturase, phage and repetitive family 13E12 genes, and INDELs (insertions and deletions) (Snps_pipeline; available in github – see below). Read sets with more than 15% of polymorphic positions classified as heterogeneous (possibly from mixed-strain cultures or strand-biased heterogeneous base calling) were excluded from downstream analyses. After sequencing quality checks, a total of 1,969 *M. bovis* genomes were selected for final analyses (Supplementary Figure S1).

### Phylogenetic Reconstruction

A customized script in Python language version 3 (snp_matrix.py; available in github – see below) was developed to build a matrix of the polymorphic positions identified in all *M. bovis* genomes. Five representative genomes of each *M. caprae* and *M. orygis* (Supplementary Table S2) (Casali et al., 2014; Broeckl et al., 2017; Marcos et al., 2017) were selected to serve as outgroup for phylogenetic inference, with corresponding reads being processed in the same manner described above for *M. bovis* genomes. *Mycobacterium caprae* and *M. orygis* have been described as the closest phylogenetic relatives to *M. bovis* (Brites et al., 2018).

For maximum likelihood (ML) phylogeny estimation, we performed ascertainment bias correction (ASC) using the “-fconst” directive of IQ-Tree. This correction is needed because SNP-based alignments overestimate branch lengths, possibly inducing biases such as inaccurate phylogeny (Lewis, 2001; Leaché et al., 2015), and overestimating clock rate priors (consequently underestimating divergence times). This was achieved by adding extra constant sites proportional to base frequencies of the MTBC using the “-fconst” option (A:17.2%, C: 32.7%, G: 32.9%, T: 17.2%, – averaged values obtained from reference assembled genomes of species available in GenBank). The resulting correction assumed absolute base counts relative to a total of 4 Mb minus the SNP alignment size, corresponding to the *M. bovis* genome size after discarding unsuitable genomic regions as described above.

The ModelFinder program (Kalyaanamoorthy et al., 2017) implemented in IQ-TREE (Nguyen et al., 2015) was used to select the optimal substitution model for the ASC-corrected SNP alignment according to Bayesian Information Criterion (BIC), including tests for discrete gamma (+G) and FreeRate (+R) models for the rate heterogeneity across sites. The optimal chosen model, TVM + F + R7, was then fixed for ML phylogenetic reconstruction. Briefly, “TVM” refers to an unequal base frequency model with different transversion rates and equal transition rates^2^, “F” means empirically counted frequencies from the alignment, and “R7” refers to the FreeRate mixed-model with seven site rate categories (Le et al., 2012; Soubrier et al., 2012). The R[*n*] model is a non-parametric way of attributing probabilities of each site pertaining to each of the *n* rate bins, accommodating site rates into a non-parametric distribution, instead of the classic gamma distribution model (+G) that has a more limited range of shapes (Yang, 1994). ML was performed using 1,000 UFBoot pseudoreplicates (a less biased ML branch support measure compared to the standard bootstrap; Minh et al., 2013; Hoang et al., 2017) in IQ-TREE, a program achieving superior ML scores in real datasets when compared to other likelihood-based programs (Zhou et al., 2017).

For further sensitivity analysis of the relevant clades and their associations found in ML, a maximum parsimony (MP) inference was employed in TNT (“Tree Analysis Using New Technology”), which is fast and accurate at estimating trees with minimum number of steps (Goloboff et al., 2008). We used an intensive (but slower) search for both MP inference and support values in TNT. A total of 500 bootstrapped datasets were run to assess MP branch support. Of note, we have also tried MPBoot (Hoang et al., 2017), which in general leads to less biased MP support values (akin to UFBoot for ML), but the program crashed even after many attempts.

Statistical assessment of the significance of ML and MP trees was performed using the approximately unbiased test (AU test), which allows comparison of multiple trees obtained using different phylogenetic methods (Shimodaira, 2002), in case there were multiple equally parsimonious MP topologies. A visual comparison of the trees was done to facilitate detection of clade differences, using the R library dendextend (Galili, 2015). Phylogenies were annotated using FigTree v1.4.3^3^ and/or Iroki (Moore et al., 2019).

### Subsampling of *M. bovis* Genomes

As to remove redundancy and have a more even representation of *M. bovis* genomes for phylogenetic description and graphical display, Treemmer software (Menardo et al., 2018) was applied in the ML phylogenetic tree containing the 1,969 *M. bovis* genomes using the parameter -RTL.95. The resulting reduced *M. bovis* dataset (*n* = 1,201), which kept 95% of the original ML tree length, was then used in downstream analysis. Another subsampling was also imposed for molecular dating (see below). In both cases, the program snp-sites (Page et al., 2016) was used to discard SNP sites that became constant after excluding the unused tips.

### SNP Markers

Unique SNPs were searched using the generated SNP matrix described above. A customized script based on Python language version 3.6.3 (snp_marker.py; available in github – see below) was developed to retrieve unique polymorphisms of each selected lineage group or cluster in the matrix (i.e., polymorphisms not present in any other lineage). SNP annotation was based on *M. bovis* AF2122/97 (NC_002945.4). Predicted proteins associated with SNP markers of *M. bovis* lineages and unknown clusters were then classified with EggNOG (Huerta-Cepas et al., 2016) into Clusters of Orthologous Groups (COG) using a graph-based unsupervised clustering algorithm extending the COG methodology (Tatusov et al., 1997). The same proteins were also analyzed in STRING database version 11.0 with default settings for the prediction of network associations between proteins (Jensen et al., 2008).

### Spoligotyping and Clonal Complexes

Spoligotypes of all selected genomes were investigated using SpoTyping (Xia et al., 2016). Identified genetic spacers were processed in the *Mycobacterium bovis* Spoligotype Database^4^ to retrieve spoligotype pattern and SB number. The following markers were used to identify clonal complexes: RdEu1 (European 1 - Eu1, RD17, 806 bp), SNP in the *guaA* gene (European 2 - Eu2), RDAf1 (African 1 - Af1, 5,322 bp) and RDAf2 (African 2 - Af2, 14,094 bp) (Müller et al., 2009; Berg et al., 2011; Smith et al., 2011; Rodriguez-Campos et al., 2012). A customized script (Snps_pipeline; #check depth in RD positions; available in github – see below) was used to detect RDEu1, RDAf1 and RDAf2 in all *M. bovis* genomes by checking their sequencing coverage when mapping each read set against the reference genome of *M. tuberculosis* H37Rv using Burrows-Wheeler Aligner (Li and Durbin, 2010). The following positions based on *M. tuberculosis* H37Rv reference genome were used: RDEu1 (1,768,074-1,768,878), RDAf1 (665,042-668,394), and RDAf2 (680,337-694,429). A region was considered deleted when average coverage was below four for RDEu1, and two for RDAf1 and RDAf2.

### Phylogenetic Clustering and Principal Component Analysis

Phylogenetic clustering was performed using TreeCluster software (Balaban et al., 2019), which is a phylogeny-based clustering method. The ML phylogenetic tree of the reduced *M. bovis* dataset (*n* = 1,201) was used as input and the method “Avg Clade” was applied, which means that the average pairwise distance between leaves in a cluster should be at most “*t*”, where we used *t* = 0.012 substitutions/site. This value of “t” was chosen after trial and error, as lower and higher values returned either undefined values for some clusters (“−1”), or an excessively low or high number of clades (in the latter case, many without clear genomic synapomorphies, precluding a biologically sound discussion of clusters). The parameter of *s* = 95 was also used, which means that leaves cannot be clustered if they are connected by branches with support less than or equal to 95%.

Principal Component Analysis (PCA) based on the generated SNP matrices of the complete (*n* = 1,969) and reduced (*n* = 1,201) *M. bovis* datasets was used to decrease their dimensionality, while allowing graphical evaluation of pairwise distances between *M. bovis* genomes. We employed the function *princomp* in R software 3.5.0 (Mardia et al., 1979; Venables and Ripley, 2002) to generate 2- and 3-dimensions PCA graphs from the SNP matrices, and dots corresponding to individual *M. bovis* genomes were colored based on pre-established lineages or clusters.

### Dating Divergences

Prior to estimating divergence times, TempEst (Rambaut et al., 2016) was used to test for tip-dating informativeness, by including all tips with bacterial isolation date available from GenBank in the ML tree (and trimming all others from the tree using the R *ape* library; Paradis et al., 2004). No significant regression (or correlation) of isolation dates to root-to-tip distances was observed (corr. = 0.06; *R*^2^ = 0.004), hence we did not use this information in further analyses. We opted for a node-dating approach (Drummond and Bouckaert, 2015) based on previous paleopathological data (Bos et al., 2014) to calculate dating estimates, as outlined below.

Molecular dating of divergences was performed using BEAST v1.10.4. (Suchard et al., 2018). We applied a Bayesian skyline coalescent tree prior, the HKY + G substitution model (for better Markov Chain Monte Carlo – MCMC – convergence when compared to the General Time Reversible – GTR – model), with a discretized gamma distribution of rates across sites with four categories, uncorrelated lognormal rates across branches, and employing calibration priors for evolutionary rates and divergence dates (described below), from here on called the “standard run.” Because we wanted to focus on a sensitivity analysis of the time and rate ranges reported by runs with different parameters, we selected a workable number of genomes (tips) from the ML tree. A total of 54 tips spanning inferred lineages were selected to facilitate MCMC convergence. Briefly, two tips (for the smaller clades) or three from each lineage were chosen. The sample emerging from each lineage’s basal node was always kept, to minimize biases on the time to most recent common ancestor (tMRCA) of that clade; and the remaining one (or two) tips were chosen randomly within each lineage, with the aid of the R *ape* library (Paradis et al., 2004). Representative genomes of phylogenetically closely related animal MTBC (*M. pinnipedii*, *M. caprae*, *M. orygis*, *M. microti*) (Brites et al., 2018) were also included in the alignment to allow calibration priors at the *M. pinnipedii* node and dating of *M. bovis* origin (Supplementary Table S2) (Bos et al., 2014; Casali et al., 2014; Marcos et al., 2017; Menardo et al., 2018; Riojas et al., 2018; Borrell et al., 2019), as further explained below. The induced 54-tip tree (counting also *M. caprae*, *M. orygis*, *M. microti*, and *M. pinnipedii* genomes) was kept fixed throughout all BEAST runs. Ascertainment bias correction of this downsized SNP alignment (10,100 polymorphic positions) was performed by adding extra constant sites proportional to base frequencies of the MTBC, as explained above, by manually editing the BEAST’s XML file.

Molecular rates of evolution were set within the range of a Uniform [1 × 1e-9; 2 × 1e-7] s/s/b/y (substitutions/site/branch/year), encompassing rates from previous studies (Ford et al., 2011; Pepperell et al., 2013; Eldholm et al., 2015; Kay et al., 2015; Lillebaek et al., 2016; Menardo et al., 2019). The minimum age was set to 800 years BP (before present) for the node marking the origin of *M. pinnipedi* (i.e., its splitting from *M. microti*), after reasonable carbon-dating skeleton data, and to a soft upper maximum of ∼7,000 years BP (a conservative upper bound), both based on Bos et al. (2014) posterior estimates, under a negative exponential prior. Such a soft upper bound allows older dates in our analyses, if these are likely to occur in the posterior distribution of our estimates in the standard run, therefore reducing the possibility of underestimating the actual time range.

Different scenarios were tested against the standard run, each changing one parameter at a time: a strict clock (instead of uncorrelated lognormal rates across branches); different tree priors (constant population, exponential growth, or birth-death with incomplete sampling, instead of the Bayesian skyline); and a different substitution model (GTR instead of HKY), for a total of six different scenarios. Model comparison was done using AICM, for its accuracy for larger alignments (Zarza et al., 2018), albeit in small alignments it tends to be inconsistent (Baele et al., 2012a, b). AICM was used instead of the stepping-stone procedure (Baele et al., 2012a, b) due to underflow issues in some of the MCMC runs.

A total of two runs were executed for each scenario of the sensitivity analysis, each with 1 billion generations (which led to convergence for all MCMC runs). Convergence and effective sample sizes (ESSs) were monitored in Tracer v1.7 (Rambaut et al., 2018), logcombiner (within the BEAST package) was used to join results from the two runs, and treeannotator (also within the same package) was used to construct the time tree after disregarding the burnin region from each run. AICM averaged values (from the two runs for each scenario) were obtained in Tracer v1.6^5^.

### Code Availability

All customized codes used in this study can be found at the github repository: https://github.com/LaPAM-USP/Zimpel-2019.

## Results and Discussion

### Phylogenetic Reconstruction of *Mycobacterium bovis* Genomes

**FIGURE 1:**
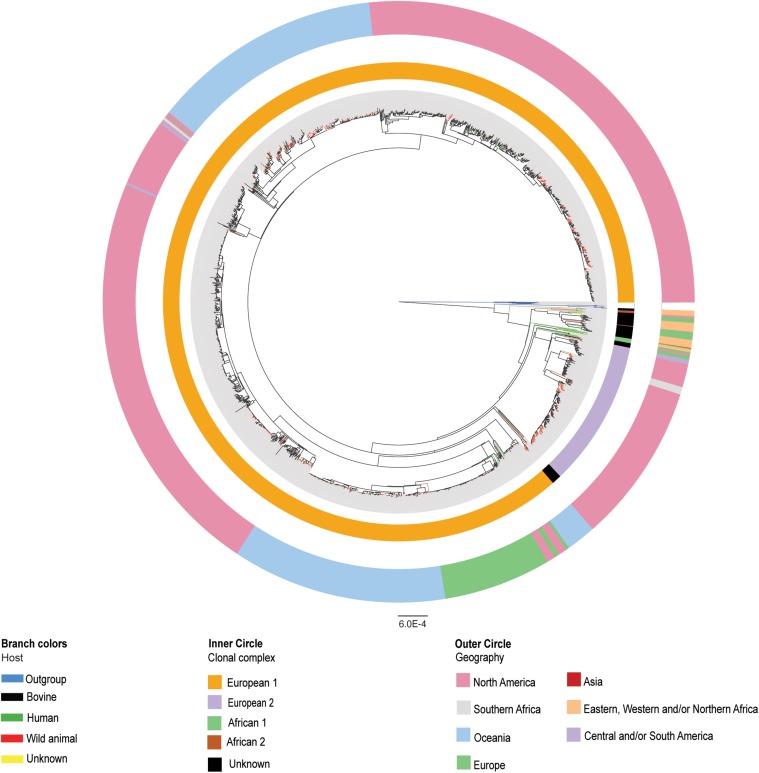
Phylogenetic reconstruction of the complete dataset of *Mycobacterium bovis* genomes (*n* = 1,969). Maximum likelihood phylogenetic tree based on single nucleotide polymorphisms (SNPs) of 1,969 *Mycobacterium bovis* genomes, using *Mycobacterium caprae* and *Mycobacterium orygis* as outgroup. The host species are marked in black branches for bovine, red branches for wild animals, green branches for humans, and yellow branches for unknown host identification. Outgroup members are marked in blue branches. Clonal complexes (inner circle) are marked in orange for European 1 (Eu1), purple for European 2 (Eu2), green for African 1 (Af1), brown for African 2 (Af2), and black for unknown. Geographical origin (outer circle) of *M. bovis* isolates are marked in pink for North America, gray for Southern Africa, blue for Oceania, purple for Central and/or South America, red for Asia, orange for Eastern, Western and/or Northern Africa, and green for Europe. Phylogenetic tree was generated using IQ-Tree with 1,000 bootstrap replicas and annotated using Iroki (Moore et al., 2019) and Adobe Illustrator. Bootstrap replicas of main nodes are all ≥90% and can be visualized in Supplementary Figure S2. Bar shows substitutions per nucleotide.

**FIGURE 2:**
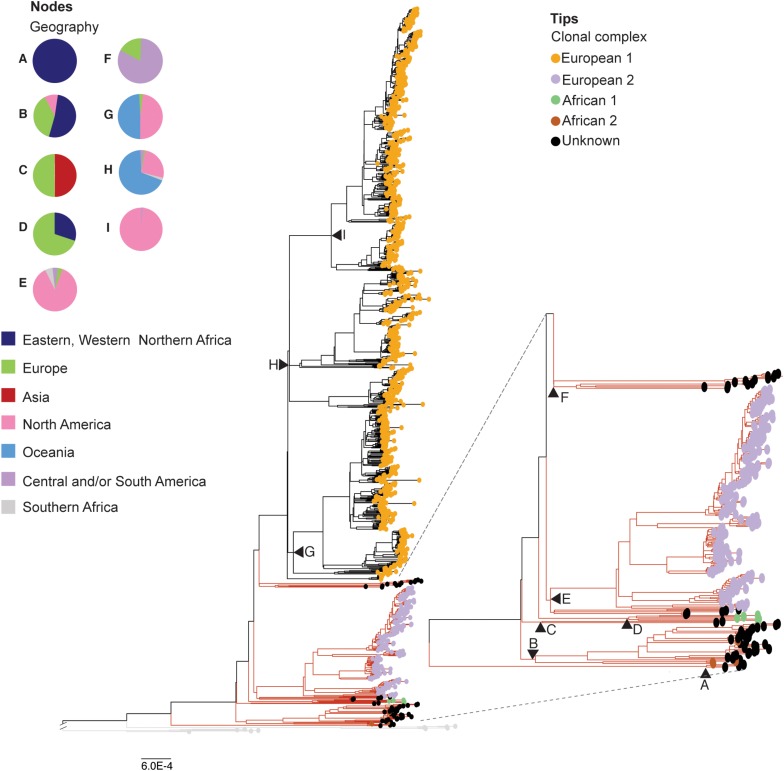
Phylogenetic reconstruction of the reduced dataset of *Mycobacterium bovis* genomes (*n* = 1,201). Maximum likelihood phylogenetic tree based on single nucleotide polymorphisms (SNPs) of 1,201 *Mycobacterium bovis* genomes, using *Mycobacterium caprae* and *Mycobacterium orygis* as outgroup. Phylogenetic tree colored based clonal complexes (CC) at the tips. Clonal complexes (circled tips): European 1 (Eu1) in orange, and European 2 (Eu2) in purple, African 1 (Af1) in green, African 2 (Af2) in brown, Unknown CC in black. Black triangles: main nodes A-I, with corresponding geographic origin of *M. bovis* genomes emerging from each node depicted by the pie charts. Branch lengths of outgroups (in light gray branches) were reduced to improve visualization of the figure. Red branches indicate a subtree that was magnified to improve visualization. Phylogenetic tree was generated using IQ-Tree with 1,000 bootstrap replicas and annotated using Iroki (Moore et al., 2019) and Adobe Illustrator. Bootstrap replicas of discussed nodes are all ≥ 95% and can be visualized in Supplementary Figure S2. Bar shows substitutions per nucleotide.

**FIGURE 3:**
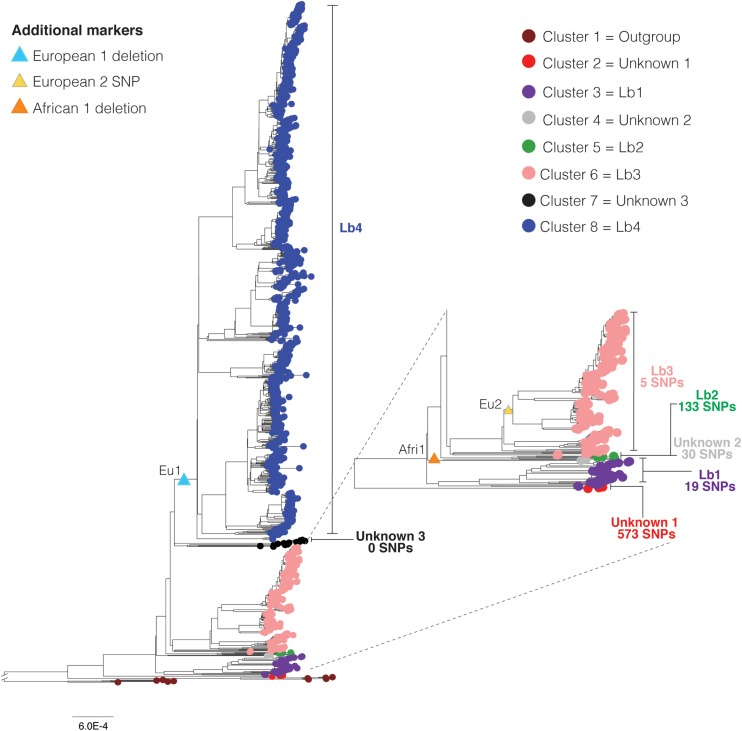
Phylogenetic clustering, SNP (single nucleotide polymorphism) markers, and lineages of *Mycobacterium bovis*. Maximum likelihood phylogenetic tree based on SNPs of 1,201 *Mycobacterium bovis* genomes (reduced dataset), using *Mycobacterium caprae* and *Mycobacterium orygis* as outgroup. Deletions and SNP correspond to the clonal complex markers European 1 (Eu1), European 2 (Eu2) and African 1 (Af1), indicated by triangles. Seven *M. bovis* clusters (2 through 8) were identified using TreeCluster (Balaban et al., 2019) and they are indicated by colored tips. Cluster 1 corresponds to the outgroup composed of *M. orygis* and *M. caprae* genomes. Cluster 1 was reported by TreeCluster as being two separated clusters (one for *M. orygis* and one for *M. caprae* genomes). In this figure we colored these clusters as one to facilitate visualization and understanding. Clusters 2–8 correspond to *M. bovis* genomes. Vertical bars indicate the number of unique SNPs in each cluster. Sixty-eight unique SNPs were also found among *M. bovis* genomes of the Eu2 clonal complex. The cluster “unknown 3” shares 33 SNPs with clusters 2, 3, 4, 5, and 6. Branch lengths of outgroups were reduced to improve visualization of the figure. Subtree is depicted separately to improve its visualization. Phylogenetic tree was generated using IQ-Tree with 1,000 bootstrap replicas and annotated using Iroki (Moore et al., 2019) and Adobe Illustrator. Bootstrap replicas of discussed nodes are all ≥90% and can be visualized in Supplementary Figure S2. Bar shows substitutions per nucleotide.

After screening ∼2,600 publicly available *M. bovis* genomes using pre-determined inclusion criteria, a total of 1,969 virulent *M. bovis* genomes from 23 countries and at least 24 different host species were selected for this study (Table 1). This sample constitutes the most comprehensive global *M. bovis* dataset ever analyzed, and includes 1,806 (91.72%) genomes published previously, and four newly sequenced genomes from this study (Supplementary Table S2). For the other 159 *M. bovis* genomes we did not find associated publications; these genomes were all sequenced by the National Veterinary Services Laboratories of the United States Department of Agriculture (USDA). We used 26,318 nucleotide polymorphic positions detected in these genomes (including outgroups, for a final alignment of 1,979 genomes) to construct phylogenetic trees inferred with maximum likelihood (ML) and maximum parsimony (MP) methodologies (Figure 1 and Supplementary Figures S2, S3). Both phylogenetic methods employed (ML and MP) generated highly similar trees (*p* ≤ 0.05) according to AU-test in IQ-TREE. Furthermore, it can be observed that the phylogenetic relationship among inferred clades is identical, with only minor changes in the positioning of subclades (Supplementary Figures S2–S4). Therefore, results are shown for ML only (Figures 1–3).

**TABLE 1:** *Mycobacterium bovis* genomes selected for analysis.

**Country**	**Number of genomes**	**Host species (# of selected read sets)**
Brazil	5	Cattle (1), Bison (1), Llama (2), Capybara (1)
Canada	7	Cattle (5), Bison (1), Elk (1)
Eritrea	13	Cattle (13)
Ethiopia	2	Cattle (2)
France	2	Cattle (1), Wild boar (1)
Germany	6	Human (6)
Ghana	3	Human (3)
Great Britain	12	Human (11), Cattle (1)
Italy	2	Human (2)
Malawi	3	Human (3)
Mexico	425	Cattle (407), Human (17), Cheese (1)
New Zealand	510	Cattle (303), Ferret (71), Pig/Porcine (16), Cervid* (15), Stoat (2), Brushtail possum (76), Feline* (2), not reported (25)
Northern Ireland	116	Cattle (112), Badger (4)
Panama	3	Cattle (3)
Russia	1	Human (1)
South Africa	11	Cattle (1), Lion (2), Kudu (1), Buffalo (7)
Spain	7	Cattle (7)
Switzerland	4	Not reported (4)
Tanzania	2	Chimpanzee
Tunisia	1	Human (1)
Uganda	1	Chimpanzee (1)
United States	828	Cattle (632), Bobcat (1), Coyote (1), Human (3), Cervid* (133), *Felis catus* (8), Elephant (1), Raccoon (12), *Sus scrofa* (6), Opossum (9), Jaguar (1), Bison (1), Elk (5), Non-human primate* (1), not reported (14)
Uruguay	5	Cattle (5)
**Total**	1,969	At least 24 host species

*Animal species not reported. *Felis catus*, domestic cat. *Sus scrofa* and pig/porcine, not reported if domestic or wild.

In the generated phylogenetic tree, host classes (bovine, wildlife and human) are found dispersed among different clades (Figure 1 - colored branches), while most *M. bovis* genomes cluster according to specific geographic locations and clonal complexes (Figure 1). This finding suggests that geographic proximity between wildlife and bovine hosts and their contact rates has played a more important role in determining host range of *M. bovis* than phylogenetic distance among hosts. The lack of host clustering supports the hypothesis of *M. bovis* being a generalist member of MTBC, able to infect different host species irrespective of the bacterial genetic makeup. Interestingly, all *M. bovis* genomes originating from the African Continent, except for South Africa, appeared as originating from relatively older nodes of the phylogenetic tree (Figure 1).

It was possible to observe an over-representation of *M. bovis* genomes from four countries: United States (828/1,969; 42.05%), New Zealand (510/1,969; 25.90%), Mexico (425/1,969; 21.58%), and Northern Ireland (116/1,969; 5.89%). Many *M. bovis* genomes originating from each of these countries formed clusters of highly similar genomes, representing redundancy in the dataset (possibly from densely sampled TB outbreaks). Therefore, we applied Treemmer software (Menardo et al., 2018) to remove redundancy while keeping 95% of the original tree length, i.e., without losing meaningful genetic diversity in the dataset. After removal, we carefully checked to guarantee that all countries were still represented and that all *M. bovis* genomes from small clades were still part of the dataset. The resulting reduced *M. bovis* dataset/tree was composed of 1,201 representative *M. bovis* genomes (Figure 2) and was used in downstream analyses.

### Clonal Complexes Do Not Represent the Whole Diversity of *M. bovis* Genomes

The distribution of CCs onto the phylogenetic trees (original and subsampled) is shown in Figures 1, 2. Accordingly, 53 out of the 1,969 (2.69%) analyzed *M. bovis* genomes do not have genetic markers of any of the four CCs. The remaining *M. bovis* isolates were found to cluster according to their CC classification. The majority (36/53, 67.92%) of *M. bovis* genomes not classified within CCs appeared dispersed, emerging from relatively ancestral nodes (Figure 1). This finding suggests that extant isolates clustering in ancient nodes were probably not included in prior studies evaluating CCs and have thus been poorly studied until now.

To facilitate data presentation and interpretation, we describe below the CC classification and country of origin of the analyzed *M. bovis* isolates in order from the most ancient to the most recent nodes of the phylogenetic tree depicted in Figure 2, generated using the reduced *M. bovis* dataset (*n* = 1,201). Accordingly, three *M. bovis* isolates from Malawi, without any CC marker, emerged from the most basal node (node A) of the phylogenetic tree (Figure 2; node A, black tips). Long branches separating these genomes from all other *M. bovis* genomes are supportive of highly divergent sequences. The subsequent *M. bovis* cluster is composed of four *M. bovis* isolates of the CC Af2 (Figure 2; isolates emerging from node B with brown tips) emerging from the node B of the phylogenetic tree together with 30 *M. bovis* genomes without CC classification (Figure 2 – isolates emerging from node B with black tips). These four *M. bovis* genomes of CC Af2 comprise one isolate from a wild boar in France, one isolate from a chimpanzee in Uganda, and two isolates from chimpanzees in Tanzania. The 30 closely related *M. bovis* genomes without clonal complex classification were obtained in Africa [Eritrea (*n* = 13), Ethiopia (*n* = 2), Tunisia (*n* = 1)], Europe [Italy (*n* = 2), Spain (*n* = 7), Switzerland (*n* = 2)], and the United States (*n* = 3). Emerging from the subsequent node (node C) of the phylogenetic tree, there are two *M. bovis* genomes obtained from human hosts in Germany and in Russia without any CC marker (Figure 2, isolates emerging from node C with black tips). These *M. bovis* genomes are located in between clades containing all genomes with markers of African CCs (Af2 and Af1), because the following clade, emerging from node D, is composed of five *M. bovis* isolates classified as CC Af1. Genomes of CC Af1 were obtained in Africa [Ghana (*n* = 3; humans)] and Europe [Germany (*n* = 1; human), Switzerland (*n* = 1; host not reported)] (Figure 2 – isolates emerging from node D with green tips).

To date, *M. bovis* strains of the CCs Af1 and Af2 have only been described in Africa (Berg et al., 2011; Firdessa et al., 2013). The presence of both African CCs in European countries, infecting a wild boar in (France), humans (Germany) and unknown hosts (Switzerland), is interesting and warrants further investigation into the actual origin of these isolates. Thirteen out of the 44 *M. bovis* genomes (29.54%) described in the paragraph above originated from isolates obtained from humans, being seven from Africa (Ghana = 3; Tunisia = 1; Malawi = 3), four from Europe (Germany = 2; Italy = 2), one from Russia, and one from the United States. Unfortunately, demographic characteristics of these patients are not described in the related literature (Casali et al., 2014; Walker et al., 2015; Brites et al., 2018; Kandler et al., 2018). With current bovine TB control, the zoonotic transmission of *M. bovis* in Europe and the USA is considered rare. Thus, we speculate that European and North American cases in humans may have arisen from zoonotic transmission acquired in the past that appears in elderly people (Majoor et al., 2011; Davidson et al., 2017) and/or imported human cases of zoonotic TB from countries where bovine TB is highly endemic.

The phylogenetic tree in Figure 2 also shows 183 *M. bovis* genomes carrying the SNP marker of the CC Eu2 (Figure 2 – purple tips, part of isolates emerging from node E). These isolates were obtained from the Americas [Brazil (*n* = 5), Canada (*n* = 2), Mexico (*n* = 33), United States (*n* = 135)], Africa [South Africa (*n* = 7)], and Europe [Germany (*n* = 1)]. There were only five *M. bovis* genomes without CC classification obtained from humans in Germany (*n* = 3) and from cattle in the United States (*n* = 2) that appeared closely related to genomes of the CC Eu2 (Figure 2 – isolates emerging from node E with black tips). The CC Eu2 has been described as dominant in the Iberian Peninsula and also detected in other countries of Europe and in Brazil (Rodriguez-Campos et al., 2012; Zimpel et al., 2017a). Its actual worldwide occurrence, however, is unknown.

The majority of the genomes in the dataset (956/1,201, 79.60%) were classified as Eu1 (i.e., having the deletion RDEu1) and originated from the Americas [USA (*n* = 357), Mexico (*n* = 354), Uruguay (*n* = 5), Canada (*n* = 5), Panama (*n* = 3)], Oceania [New Zealand (*n* = 214)], Europe [Great Britain (*n* = 8), Northern Ireland (*n* = 6)] and Africa [South Africa (*n* = 4)] (Figure 2 - isolates emerging from nodes G, H and I with orange tips). Interestingly, *M. bovis* of the CC Eu1 have been found to be dispersed worldwide (Smith et al., 2011); in our analysis they emerged from the most recent evolutionary node of the phylogenetic tree. There were also 13 genomes closely and basally related to *M. bovis* genomes of CC Eu1 that did not carry any CC marker and were obtained in Europe [France (*n* = 1; cattle), Great Britain (*n* = 1; human)] and the United States (*n* = 11; seven from cervids, one from a non-human primate, one from an elephant, and two from cattle) (Figure 2 – isolates emerging from node F with black tips).

### Phylogenetic Clustering

It has been recently shown that phylogeny-based clustering of populations can better reflect divergence and similarity between microorganisms when compared to more simplistic pairwise distance-based methods (Ragonnet-Cronin et al., 2013; Balaban et al., 2019). By taking into account phylogenetic trees, phylogeny-based clustering methods leverage from model-based, statistically rigorous corrections of sequence distances depicted in branch lengths (Balaban et al., 2019). Thus, we used TreeCluster, a phylogeny-based clustering method, to better understand the populational structure of *M. bovis* genomes. Accordingly, seven distinct clusters of *M. bovis* were detected using the ML phylogenetic tree of the reduced dataset of *M. bovis* genomes (*n* = 1,201) as input to the algorithm (Figure 3 – colored tips; clusters 2–8), circumscribing clusters that could be in general associated with SNP markers (see below) and clonal complexes.

### SNP Markers

We used the generated SNP matrix of the 1,201 *M. bovis* genomes (reduced dataset) to search for SNPs that were unique to specific groups of genomes of the phylogenetic tree (i.e., present in 100% of the strains in the analyzed group and not present in strains outside of that group) (Figure 3 and Supplementary Table S3). Explaining the long divergent branch, a total of 573 SNPs were found to be unique in *M. bovis* genomes from Malawi (Figure 3 – cluster 2, in red). Nineteen SNPs were found to be unique to the group comprising the four genomes of *M. bovis* classified as CC Af2 and the 30 closely related *M. bovis* genomes without CC classification (i.e. the group originating from node B of the phylogenetic tree in Figure 2) (Figure 3 – cluster 3, in purple). Therefore, these 19 SNPs are more stable markers of this group than the RDAf2 deletion. On the other hand, 133 SNPs were found to be unique to the small group of five *M. bovis* genomes with the RDAf1 deletion, further supporting the segregation of this phylogenetic group (Figure 3 – cluster 5, in green). Thirty SNPs were found to be unique of the *M. bovis* genomes from Russia and Germany without CC markers, in between Af1- and Af2-related clusters (Figure 3 –cluster 4, in light gray).

In addition to the SNP in *guaA* gene that determines the CC Eu2, 68 SNPs were found to be unique to *M. bovis* genomes classified as CC Eu2 in this study (Figure 3 – part of cluster 6, emerging from the node marked with Eu2 SNP, yellow triangle). We also found 5 SNPs that were unique to the *M. bovis* of CC Eu2 and the five closely related *M. bovis* genomes without CC classification, supporting the genetic segregation of this phylogenetic group (Figure 3 – cluster 6, in pink).

There were no SNP markers unique to *M. bovis* of the CC Eu1; the only stable genetic marker is the deletion RDEu1 (Figure 3 – cluster 8, in blue). The 13 genomes without CC classification that are closely related to *M. bovis* genomes of CC Eu1 did not share unique SNPs among them and with genomes of the CC Eu1 (Figure 3 – cluster 7, in black). Interestingly, these genomes shared 33 unique SNPs with the most basal genetic groups of the phylogenetic tree (clusters 2, 3, 4, 5, and 6). Genomic position and annotation of SNPs are reported in Supplementary Table S3, while COG and STRING analyses of affected genes are reported in Supplementary Figures S8–S13. Future studies should be conducted to determine the impact of these mutations, particularly if non-synonymous, on the functionality of the corresponding proteins and bacterial phenotypes of different *M. bovis* clusters.

### A Proposal for Global Lineages of *M. bovis*

Based on the observed tree topology, CC distribution, geography, phylogenetic clustering, and SNP markers, we propose the existence of at least four major global lineages of *M. bovis*, which we define as Lb1, Lb2, Lb3, and Lb4 (Figure 3 and Table 2). These lineages were either exclusively composed of *M. bovis* genomes with specific CC markers (Lb2 and Lb4; Figure 3 – Eu1 and Af1) or composed of a mixture of *M. bovis* genomes carrying a CC marker or not (Lb1 and Lb3; Figure 3). We also identified three small clusters (unknowns 1, 2, and 3; Figure 3) not associated with any CC marker and composed of few genomes (3, 2, and 13, respectively; Figure 3).

**TABLE 2:** Distribution of *Mycobacterium bovis* lineages in the studied countries.

**Country**	**Lb1**	**Lb2**	**Lb3**	**Lb4**	**Unknown 1**	**Unknown 2**	**Unknown 3**
Malawi	–	–	–	–	3	–	–
Eritrea	13	–	–	–	–	–	–
Ethiopia	2	–	–	–	–	–	–
Tanzânia	2	–	–	–	–	–	–
Uganda	1	–	–	–	–	–	–
Tunisia	1	–	–	–	–	–	–
France	1	–	–	–	–	–	1
Germany	–	1	4	–	–	1	–
Spain	7	–	–	–	–	–	–
Italy	2	–	–	–	–	–	–
Russia	–	–	–	–	–	1	–
Ghana	–	3	–	–	–	–	–
Switzerland	2	1	–	–	–	–	–
United States	3	–	137	357	–	–	11
Mexico	–	–	33	354	–	–	–
Canada	–	–	2	5	–	–	–
Brazil	–	–	5	–	–	–	–
South Africa	–	–	7	4	–	–	–
Northern Ireland	–	–	–	6	–	–	–
Great Britain	–	–	–	8	–	–	1
New Zealand	–	–	–	214	–	–	–
Panama	–	–	–	3	–	–	–
Uruguay	–	–	–	5	–	–	–
Total *	34	5	188	956	3	2	13

Lineage Lb1 is composed of 34 *M. bovis* isolates, encompassing the four representatives of CC Af2 and the 30 closely related genomes without CC classification (Figure 3 - in purple). We provisionally propose the 19 above-described unique SNPs as identification markers for this lineage (Supplementary Table S3). The observed geographical origin of extant Lb1 isolates points toward North and East Africa and Europe, although it can also be detected, at a lower frequency, in the United States (three Lb1 isolates out of 503 *M. bovis* isolates from the United States) (Table 2). Lb2 lineage (Figure 3 – in green) is composed of the five *M. bovis* genomes of CC Af1. These genomes are segregated by the RDAf1 deletion and 133 SNP markers. As additional *M. bovis* genomes of this linage are sequenced in the future, we expect the robust establishment of RDAf1 as a stable marker and the refinement of the number of unique SNP markers. Presently, the extant Lb2 strains comprise isolates from Ghana (*n* = 3), Germany (*n* = 1) and Switzerland (*n* = 1) (Table 2). Unfortunately, host information was not available for the isolate obtained in Switzerland. It is possible that this *M. bovis* strain was isolated from a human patient, which precludes geographical origin analyses due to human migration. Nevertheless, both extant Lb1 and Lb2 strains have strong ties to North, East and West Africa and to a lesser degree with Europe and the United States.

Lb3 is composed of the 183 *M. bovis* of CC Eu2 and the five genomes without CC classification (Figure 3 - in pink), being supported by five unique SNPs (Supplementary Table S3). Interestingly, *M. bovis* genomes of CC Eu2 comprises a rapidly evolving and geographically diverse sublineage (Lb3.1) when compared to the three genomes obtained from humans in Germany and two genomes obtained from cattle in the USA without a CC marker (Lb3.2). Again, geographic origin and country history of these infected humans are not described in the related literature (Walker et al., 2015) and it is possible that these patients acquired the infection in the past, as described above, representing isolates of an older origin, and/or in another country. The CC Eu2 is not a stable marker for Lb3 as a whole; instead, the five unique SNPs may be used as identification markers for Lb3 (Supplementary Table S3).

Finally, lineage Lb4 (Figure 3 – in blue) is composed of 956 genomes of *M. bovis* of the CC Eu1, indicating that RDEu1 is a stable marker of this evolutionarily recent lineage. Lineage Lb4 is composed of a higher number of *M. bovis* genomes when compared to other lineages due to over-representation of geographically restricted genomes from the United States, New Zealand, Northern Ireland, and Mexico. The ladder-like tips observed in Lb4 are consistent with recent population expansion across subpopulations of Lb4, which may be directly influenced by the persistence of *M. bovis* in different wildlife populations, variations in the efficiency of bovine TB control programs and geographic isolation. The tree topology suggests that once an ancestral *M. bovis* strain was introduced into each of these countries, this subpopulation clonally expanded in geographic isolation, except for certain clusters with *M. bovis* genomes from Mexico and the United States.

With the described SNPs markers and CC deletions, it is not possible to provide robust classification for the *M. bovis* genomes shown as clusters “unknown 1,” “unknown 2,” and “unknown 3” in Figure 3. The *M. bovis* genomes from Malawi (“unknown 1”) appear as a putative ancient lineage of this bacterial species, with very divergent genome sequences represented by long branches and high number of unique SNPs. However, only three genomes are available, which can overestimate the number of unique SNPs associated with this putative lineage. It is expected that the sequencing of additional genomes from the same geographic region and the search for specific genetic markers (SNPs and deletions) in the future will provide a better opportunity to robustly characterize this lineage.

The two genomes of cluster “unknown 2” (Figure 3), from Russia and Germany, have no CC markers. In another phylogenetic tree that we generated, using fewer genomes and with *M. tuberculosis* H37Rv as an outgroup, these genomes clustered with Lb1 (data not shown). Therefore, it is possible that a systematic error of long branch attraction and/or number of genomes may interfere with the correct placement of these genomes. And finally, the 13 genomes without a CC marker closely related to Lb4 (from France, Great Britain, and the United States) (Figure 3) do not share unique genetic markers with Lb4 or Lb3 (i.e., SNPs not present in any other lineage) or among themselves. They do share, however, 33 SNPs with the more ancient lineages Lb1, Lb2, and Lb3 (Figure 3), being thus more closely related to these strains than with Lb4. Further genomes should be sequenced to better characterize this group.

### Spoligotyping Patterns Are Correlated With *M. bovis* Lineages

To further support our findings related to the global *M. bovis* lineages, we also evaluated the spoligotype of all isolates. There was a good association between spoligotype and the four *M. bovis* lineages (Supplementary Table S4), except for SB0120, SB0265 and SB1345, which appear in both Lb3 and Lb4. Thus, the vast majority of the patterns were specific to the predicted lineages, demonstrating that spoligotyping can be a supporting tool to infer these groups, albeit keeping in mind that homoplasy is a common phenomenon in spoligotypes (i.e., identical spoligotype patterns can occur independently in unrelated lineages because the loss of spacer sequences is a common event) (Warren et al., 2002) and may also occur with lineage classification (e.g., SB0120, SB0265, and SB1345 appear in Lb3 and Lb4).

### Principal Component Analysis

**FIGURE 4:**
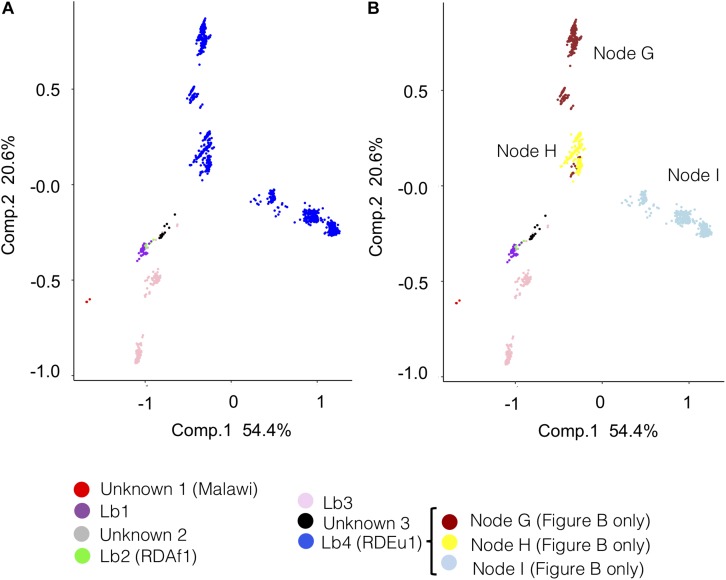
Principal component analysis (PCA) of *Mycobacterium bovis* lineages according to the reduced dataset (*n* = 1,201). The PCA graphs were constructed using the SNP (single nucleotide polymorphism) matrix of the 1,201 *Mycobacterium bovis* genomes. **(A)** PCA colored according to the proposed *M. bovis* lineages (Lb1, Lb2, Lb3, and Lb4) and unknown clusters (unknown 1, 2, and 3). **(B)** PCA colored according to the proposed lineages Lb1, Lb2, and Lb3, unknown clusters (unknown 1, 2, and 3) and clusters of *M. bovis* genomes emerging from nodes G, H, and I of Lb4 as depicted in the phylogenetic tree in Figure 2. The four inferred *M. bovis* lineages and unknown clusters are shown in purple – Lb1; green – Lb2; pink – Lb3; blue – Lb4; red – cluster unknown 1 (Malawi); gray – cluster unknown 2; black – cluster unknown 3. PCA analysis in 3 dimensions is shown in Supplementary Figure S5. PCA analyses using full dataset (*n* = 1,969) are shown in Supplementary Figures S6, S7.

To further evaluate the genetic relationship among *M. bovis* lineages, the SNP matrices of the reduced (*n* = 1,201) and original (*n* = 1,969) datasets of. *M. bovis* genomes were subjected to principal component analyses (Figure 4 and Supplementary Figures S5–S7). Results suggest a robust segregation of the modern lineage Lb4 and a close genetic relatedness of the more ancient lineages Lb1, Lb2 and Lb3. These findings directly reflect the results of the phylogenetic tree and SNP markers, as the lineages emerging from the most ancestral nodes appeared more closely related and shared 33 unique SNPs. The resulting PCA analysis is similar to what is observed using an equivalent approach with *M. tuberculosis* lineages, in which the modern lineages of *M. tuberculosis* (L2, L3 e L4) appear closely related, whereas the ancient lineages (L1, L5, L6, and L7) are found segregated from the rest, in different groups (Brites et al., 2018). Both PCA graphs (original and reduced dataset; Figure 4 and Supplementary Figures S5–S7) suggests that Lb4, which is markedly characterized by the Eu1 deletion, is likely composed of three sublineages that correspond to *M. bovis* genomes emerging from nodes G, H, I of the phylogenetic tree depicted in Figure 2. The *M. bovis* genomes emerging from node I are mostly from North America (437/439; 99.54%) (two genomes only are from Panama and Uruguay) (Figure 2), which may indicate that these Lb4 sublineages have been evolving under geographical segregation. Although two separated clusters for Lb3 were observed in the PCA generated with the reduced dataset (Figure 4 and Supplementary Figure S5), this segregation was not sustained when using the original/complete dataset (Supplementary Figures S6, S7). Our findings highlight the need to further evaluate the virulence phenotype of the proposed *M. bovis* lineages, as ancient and modern *M. tuberculosis* lineages display distinct abilities to cause disease (de Jong et al., 2010; Portevin et al., 2011).

### Dating Estimates for the Origin of *M. bovis*

Among all six dating models tested, three models fit better the dataset according to AICM, following published guidelines suggesting that ΔAICM values > 10 are sufficient to render a model unlikely (Burnham and Anderson, 2002) (Supplementary Table S5). These models are: constant population coalescent prior (Const_pop), birth-death with incomplete sampling tree prior (Birth-Death), and exponential population coalescent prior (Exp_pop). Dating results are thus presented for these three cases.

**FIGURE 5:**
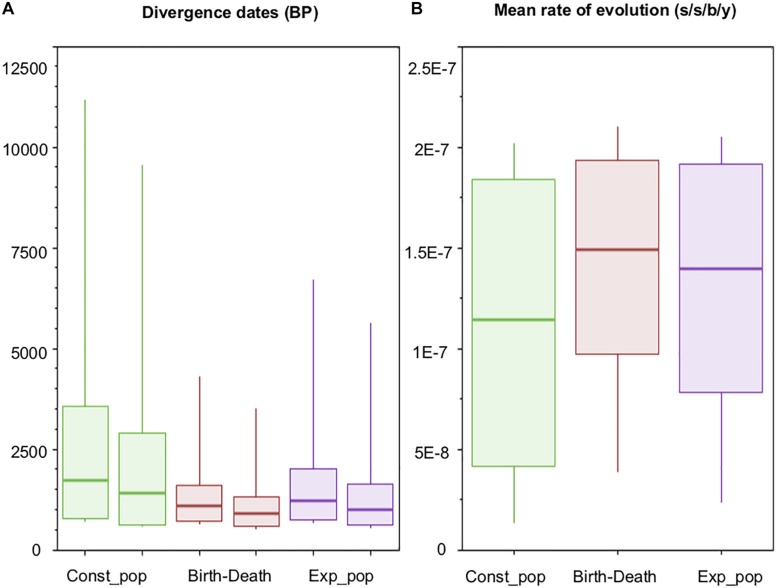
Dating estimates and molecular rates of evolution. Box-Plots showing **(A)** 95% highest posterior densities (95% HPDs) of origin and divergence times of *Mycobacterium bovis*, for each of the three models with relatively better fit (see text for details); and **(B)** 95% HPDs of the mean rate of evolution across Markov Chain Monte Carlo MCMC generations (parameter ‘meanRate’ in BEAST). s/s/b/y: substitution/site/branch/year.

Recent analyses point toward the evolutionary rate defined by Bos et al. (2014) as being the most plausible to explain the trajectory of MTBC (Menardo et al., 2019; O’Neill et al., 2019). Posterior evolutionary rates obtained herein (Figure 5B) are in agreement with the range proposed by Bos et al. (2014), even though our rate priors allowed values one order of magnitude higher or lower (see section “Materials and Methods”).

Our posterior Bayesian estimate indicates that the origin of *M. bovis* (i.e., time of the MRCA of *M. caprae* and *M. bovis*) occurred conservatively between 715 and 3,556 years BP (Before Present), taking the overall minimum date and overall maximum date from the following estimates: 782 – 3,556 years BP (Before Present) by the Const_Pop model, 715 – 1,604 years BP by the Birth-Death model, and 752 – 2,009 years BP by the Exp_Pop model (Table 3 and Figure 5A). This maximum time obtained (3,556 years BP) (Table 3), which is a time given by the Const_Pop model, agrees with the archeological finding of *M. bovis* (defined by the loss of RD4) causing infection in four human skeletons from the Iron Age in South Siberia, with carbon-dating placing them within the 1,761 – 2,199 years BP range (Taylor et al., 2007). Interestingly, these ancient *M. bovis* strains did not have the deletion marker of CC Eu1 (i.e., RD17) (Taylor et al., 2007), which is evidence that Lb4 is indeed a more recently evolved lineage of *M. bovis*.

**TABLE 3:** Dating estimates of the origin of *Mycobacterium bovis* and its lineages and clusters.

	**Estimate date divergence**
**Lineages and**	**in years before**
**clusters**	**present (BP)**
*M. bovis*	715 – 3,556
Cluster “unknown 1”	597 – 2,903
Lineage 1 (Lb1)	402 – 1,964
Cluster “unknown 2”	395 – 1,853
Lineage 2 (Lb2)	372 – 1,793
Lineage 3 (Lb3)	364 – 1,719
Cluster “unknown 3”	338 – 1,638
Lineage 4 (Lb4)	338 – 1,638

Dating estimates reported herein correspond to the origin of *M. bovis*, and not necessarily to the origin of the disease or similar clinical presentations known as bovine TB. We cannot assume for certain that the disease bovine TB originated only during this period. The disease may have been present in cattle before this time, although caused by a different ancestor of a species we presently call *M. bovis*. It is still unclear if the ancestor of MTBC was a specific human pathogen or a generalist microorganism able to infect multiple host species (Brites et al., 2018). In the latter case, the intensification of the livestock system and increase in animal and human population density may have selected for pathogens either more adapted to animals (*M. bovis*) or more adapted to human beings (*M. tuberculosis*) over evolutionary time.

It should be noted that genomes from representative *M. bovis* isolates of cattle from Asia have yet to be sequenced and analyzed. There is also a paucity in our dataset of *M. bovis* genomes from the African (*n* = 36/1,969; 1.83%) and European continents [*n* = 33/1,969; 1.68%, excluding genomes from Northern Ireland, and emphasizing that among these 33 genomes, 19 (57.57%) are from human patients of unknown origin]. The lack of information from these continents, as well as limited paleozoological data of bovine or zoonotic TB, precludes accurate estimations for the ancestral geographic origin of *M. bovis* lineages. Nevertheless, it should be highlighted that *M. bovis* genomes from Northern, Western and Eastern Africa constitute the clades emerging from the relatively older nodes of our ML phylogenetic tree (Figures 1, 2) (25 out of 36 genomes from the African continent – the remaining 11 are from South Africa). As MTBC is believed to have originated in East or West Africa (Brites and Gagneux, 2017; O’Neill et al., 2019), it is possible that this was also the case for *M. bovis*. Alternatively, the more recent emergence of *M. bovis* may have provided the opportunity of origin and diversification outside of Africa with subsequent re-introduction into that continent. Further studies with additional *M. bovis* genomes from underrepresented regions should be conducted in the future to elucidate this matter.

It is important to mention that the node-dating approach applied herein to estimate dates is not without caveats. A parametric distribution had to be assumed as prior, altogether with a maximum date that could be either under- or overestimated, and therefore posterior date estimates could be too conservative. In such cases, however, when the whole analysis is extremely dependent on a single node prior, employing sensitivity analyses of different parameters on time estimates, as well as performing model fit comparison, is essential to keep the most conservative 95% HPD (highest posterior densities), to minimize the possibility of reporting a more precise yet inaccurate range (Drummond and Bouckaert, 2015). Therefore, it is possible that our maximum estimates are overly conservative, a premise that could be further tested in light of new paleopathological data, for example.

### Bovine TB After the 16th Century

Our dating estimates and evolutionary predictions reveal a complex relationship between spatial dispersal and expansion of *M. bovis*. Bovine TB worldwide distribution is certainly influenced by import and export of cattle breeds over time. Non-European countries imported specialized breed cattle from single sources and then exported to other countries, while others imported animals from multiple locations. Estimates show that the MRCAs of extant *M. bovis* isolates occurring in Northern Ireland date between 53 and 283 years BP by the Const_Pop model, 55 and 145 years BP by the Birth-Death model, and 56 and 175 years BP by the Exp_Pop model. Archeological data from cattle specimens found in the UK (United Kingdom) indicates that these animals had a shared ancestry with wild British aurochs, being unrelated to the modern European taurine (Orlando, 2015). Accordingly, in Northern Ireland, cattle have been the mainstay of farming since the Neolithic period (about 6,000 BP). Therefore, despite the ancient presence of cattle in these regions, the introduction of the extant *M. bovis* lineage Lb4 seems to be a more recent event, possibly due to the importation of taurine cattle from other areas of Europe.

Most of the United States isolates clustered in Lb4 (357/508), a few in Lb3 (137/508) and only three in Lb1 (3/508). A similar trend is observed in Mexico, with the majority of the isolates clustered in Lb4 (354/387) and fewer representatives in Lb3 (33/387). Our dating estimates are consistent with bovine TB introduction into these countries during the New World colonization period, with Lb3 dating from 347 to 1,665 years BP by the Const_Pop model (335–1623 and 352–765 years BP by Birth-Death and Exp_Pop models, respectively), and Lb4 estimated with similar ranges (353 – 755, 365 – 954, and 360 – 939 years BP by the respective models). The first cattle to be introduced into the United States came from the Iberian Peninsula in the 16th century, being contained within our estimated date ranges. This introduction was followed by cattle importation from Mexico (which also received animals from the Iberian Peninsula) and from England (Martínez et al., 2012). This is consistent with the introduction of Lb3 (CC Eu2) and Lb4 (CC Eu1) into United States and Mexico, common lineages in the Iberian Peninsula and the United Kingdom, respectively. Isolates from United States and Mexico frequently clustered together, consistent with the close cattle trade relationship between these two countries throughout modern history.

In New Zealand, Lb4 introduction is estimated to have occurred between 78 and 494 years BP by the Const_Pop model, 89 and 246 years BP by the Birth-Death model and 85 and 297 years BP by the Exp_Pop model. Breeds of cattle were introduced into New Zealand in the 19^*th*^ century (Livingstone et al., 2015), which is within our time period range and suggests subsequent spreading to wildlife as a possibility. In contrast, *M. bovis* genomes from South Africa appeared clustered in Lb3 and Lb4 (Smith et al., 2011). Despite the small sample size, they are not present in the most ancient lineages, Lb1 and Lb2. In fact, previous genotyping studies have shown that the most common *M. bovis* CC in South Africa is Eu1, i.e., Lb4. Thus, in agreement with previous hypotheses (Michel et al., 2009; Smith et al., 2011), it is likely that bovine TB was introduced into South Africa following European colonization, and subsequently spilled over to naïve wildlife, with destructive consequences (Michel et al., 2006), as exemplified by *M. bovis* genomes from kudus, lions and buffalos analyzed herein.

## Conclusion

We propose the existence of at least four global lineages of *M. bovis*, named Lb1 and Lb2, occurring mostly in Africa and Europe, Lb3 present mainly in the Americas, Europe and South Africa, and Lb4 dispersed worldwide. Because these lineages tend to cluster based on geographical location rather than host species, it reinforces the idea that bovine TB eradication will only be attained once the disease is controlled in wildlife and vice-versa. The observed lack of host specificity supports the hypothesis of *M. bovis* being a generalist member of MTBC. Nonetheless, *M. bovis* ability to be transmitted among cattle is the main reason why this pathogen has spread geographically over evolutionary time, because of animal trade.

CC and/or SNP markers are proposed for each lineage. Our results shown that CC markers are only stable for Lb2 (Af1) and Lb4 (Eu1), while Lb1 and Lb3 can be better identified using whole genome sequencing and a provisional set of SNPs. The lack of stable CC markers in Lb1 indicates that these pathogens have been the least studied, and that there is an urgent need for additional evaluations of *M. bovis* isolates from Africa, as well as from Asia and continental Europe (given the low number of genome representatives from these continents). Further sequencing of *M. bovis* isolates throughout the world will provide the opportunity to refine the identification of SNP and deletion markers specific of each lineage, as well as provide accurate data from geographic areas not explored in this study.

Our results delineate independent evolutionary trajectories of bacterial subpopulations (i.e., lineages) of *M. bovis* underlying the current disease distribution. Whether or not these events are associated with further specialization of *M. bovis* to the bovine species and breeds or increased/decreased virulence of this pathogen in domesticated and/or wildlife have yet to be determined. Lineages of *M. tuberculosis* are known for their virulence variations, warranting further similar studies regarding *M. bovis* lineages.

Our dating analysis and molecular evolutionary rate range determination were based on a sensitivity analysis involving changes in different parameters known to affect similar studies (Drummond and Bouckaert, 2015; and references therein), such as different tree priors, evolutionary models, and molecular clock assumptions. These models were then screened for the best relative fit, rendering some reliability to the posterior estimates of the more appropriate models. Notwithstanding, even the best predicted model assumptions may not always apply to a given dataset, so comparisons to independent data can provide further evidence of the likelihood of time and rate ranges. Accordingly, our analyses agree with archeological data of *M. bovis* in Siberian carbon-dated skeletons from ∼2,000 years BP, and known times of cattle introduction into different countries (New Zealand and Mexico/United States), while also not negating introduction times in Northern Ireland. Furthermore, evolutionary rates obtained herein are also in agreement with a study based on careful examination of Pre-Columbian Peruvian mummies infected with *M. pinnipedii* that were analyzed against modern MTBC genomes (Bos et al., 2014), even though our rate priors allowed values one order of magnitude higher, and lower. Future dating analyses could test the amplitude of further assumptions (e.g., likelihood of rate-dependency across the tree, impact of positive and/or negative selection on dating estimates, usefulness and inclusion of different data types in the same analysis, among others), which are still not easily applicable to phylogenomic datasets at the present time.

Finally, dating estimates of *M. bovis* introduction into the New World, New Zealand and South Africa can be explained by the history of economic trade, especially involving animals, supporting the continuous spread of bovine TB worldwide over time. Unfortunately, the paucity of *M. bovis* genomes from Africa, Asia and continental Europe precludes the precise estimation of the geographic origin of *M. bovis*. Increasing investments in *M. bovis* genome sequencing in the future may allow such studies. By understanding the evolutionary origin and genomic diversification of *M. bovis*, we expect that the results presented herein will help pave the way to avoid future outbreaks of the disease in cattle, wildlife, and humans.

## Data Availability Statement

The datasets generated for this study can be found in the NCBI SRA accession numbers: SRR7693912, SRR7693877, SRR9850824, and SRR9850830.

## Author Contributions

CZ performed and/or participated in all experiments, analyzed and interpreted the data, and wrote the manuscript. JP designed and performed dating divergence experiments, analyzed and interpreted the resulting data. ACG performed the experiments involving the generation and analyses of the SNP matrix. RS designed and supervised the specific bioinformatics analyses. TS-P provided bioinformatic assistance for the phylogenomic analysis. NC provided bioinformatics assistance for the phylogenomic analysis. AS performed the experiments involving bacterial isolation and DNA extraction. CI performed the experiments involving bacterial isolation and DNA extraction. JN, JS, and MH designed the experiments and analyzed the data. AMG designed and coordinated the study, analyzed the data and wrote the manuscript.

## Conflict of Interest

The authors declare that the research was conducted in the absence of any commercial or financial relationships that could be construed as a potential conflict of interest.

## Footnotes

1 https://github.com/broadinstitute/picard

2 http://www.iqtree.org/doc/Substitution-Models

3 http://tree.bio.ed.ac.uk/software/figtree/

4 www.mbovis.org

5 http://beast.bio.ed.ac.uk
