# Supplementary material for: Global Distribution and Evolution of Mycobacterium bovis Lineages
Source: Front Microbiol. 2020 May 7;11:843. doi: 10.3389/fmicb.2020.00843 (PMC7232559; doi:10.3389/fmicb.2020.00843)
Supplement: Supplementary file 1 [file Table_1.DOCX]

**Table S1**. Features of read sets of *Mycobacterium bovis* initially selected for the analysis.

| **Country** | **Read sets** | **Host species (# of selected read sets)** |
| --- | --- | --- |
| Brazil | 5 | Cattle (1), Bison (1), Llama (2), Capybara (1) |
| Canada | 7 | Cattle (5), Elk (1), Bison (1) |
| China | 2 | Primate (1), Unknown host (1) |
| Eritreia | 14 | Cattle (14) |
| Ethiopia | 2 | Cattle (2) |
| France | 2 | Cattle (1), Wild boar (1) |
| Ghana | 5 | Human (5) |
| Germany | 7 | Human (7) |
| Italy | 2 | Human (2) |
| Malawi | 3 | Human (3) |
| Mexico | 463 | Cattle (445), Human (17), Cheese (1) |
| New Zealand | 515 | Cattle (305), Ferret (72), Pig (16), Deer (15) Stoat (2), Feline (2), Possum (77), Unknown host (26) |
| Northern Ireland | 150 | Cattle (146), Badger (4) |
| Panama | 9 | Cattle (9) |
| Republic of Congo | 3 | Human (3) |
| Russia | 2 | Human (2) |
| South Africa | 13 | Cattle (2), Lion (3), Kudu (1), Buffalo (7) |
| Spain | 7 | Cattle (7) |
| Switzerland | 4 | Unknown host (4) |
| Tanzania | 2 | Chimpanzee (2) |
| Tunisia | 1 | Human (1) |
| Uganda | 2 | Chimpanzee (1), Human (1) |
| United Kingdom | 24 | Human (17), Cattle (7)* |
| United States | 936 | Bovine (729), Coyote (1), Human (3), Cervid (152), Cat (8), Elephant (1), Raccoon (14), Wild boar (8), Opossum (11), Jaguar (1), Bobcat (1), Bison (1), Elk (5), Non-human primate (1) |
| Uruguay | 22 | Cattle (22) |
| **Total** | 2,202 |  |

* One genome is the reference genome *M. bovis* AF2122/97.
