# Supplementary material for: Global Distribution and Evolution of Mycobacterium bovis Lineages
Source: Front Microbiol. 2020 May 7;11:843. doi: 10.3389/fmicb.2020.00843 (PMC7232559; doi:10.3389/fmicb.2020.00843)
Supplement: Supplementary file 5 [file Table_5.docx]

**Table S5.** Akaike’s information criterion (AICM) for model selection in BEAST v 1.10.4. (Bayesian Evolutionary Analysis Sampling Trees)

| **Model** | **AICM** | **Delta AICM** |
| --- | --- | --- |
| Constant population | 10964376.62 | - |
| Birth death | 10964376.94 | 0.32 |
| Exponential population | 10964380.82 | 4.21 |
| GTR | 10964807.44 | 430.8 |
| Standard | 10964934.12 | 557.5 |
| Strict Clock | 10967317.55 | 2940.93 |
