# Supplementary material for: Global Distribution and Evolution of Mycobacterium bovis Lineages
Source: Front Microbiol. 2020 May 7;11:843. doi: 10.3389/fmicb.2020.00843 (PMC7232559; doi:10.3389/fmicb.2020.00843)
Supplement: Supplementary file 6 [file Data_Sheet_1.DOCX]

**Figure S1.** Flowchart of the selection of *Mycobacterium bovis* genomes deposited in the Sequence Read Archive (SRA) of the National Center for Biotechnology Information (NCBI) in September 2018. * Number of *Mycobacterium bovis* genomes of each country: United States of America = 936; New Zealand = 515; Northern Ireland = 150; Mexico = 463; United Kingdom = 17; Uruguay = 22; Eritrea = 14; Ethiopia = 2; France = 2; Panama = 9; Italy = 2; Brazil = 5; Canada = 7; Tunisia = 1; China = 2; Spain = 7; South Africa = 13; Uganda = 2; Tanzania = 2; Mali = 1. Total n = 2,172. ** *Mycobacterium bovis* genomes made available by Brites et al., 2018: Switzerland = 4; Ghana = 5; Malawi = 3; United Kingdom = 6; Germany = 7; Russia = 2; Republic of Congo = 3. Total n = 30.

**Figure S2.** Maximum likelihood phylogenetic tree with bootstrap values based on single nucleotide polymorphisms (SNPs) of 1,969 *Mycobacterium bovis* genomes using *Mycobacterium caprae* and *Mycobacterium orygis* strains as outgroup. Phylogenetic tree was generated using IQ-Tree (Nguyen et al., 2015) with 1,000 bootstrap values and annotated using FigTree v1.4.3 (Rambaut, 2012). Bootstrap support values ≥ 90 are shown.

**Figure S3.** Maximum parsimony phylogenetic tree with bootstrap values based on single nucleotide polymorphisms (SNPs) of 1,969 *Mycobacterium bovis* genomes using *Mycobacterium caprae* and *Mycobacterium orygis* as outgroup. Phylogenetic tree was generated using TNT (“Tree Analysis Using New Technology”) (Goloboff et al., 2005) with 500 bootstrap values and annotated using FigTree v1.4.3 (Rambaut, 2012). Bootstrap support values ≥ 90 are shown.

**Figure S4.** Comparison between maximum likelihood (ML) and maximum parsimony (MP) phylogenetic trees of the complete *Mycobacterium bovis* dataset (*n*=1,969) using the R library *dendextend* (Galili, 2015). (A) Side-by-side comparison between clusters of ML and MP according to the results of TreeCluster; fine-level clade differences (B) Tanglegram comparing ML and MP, where similar sub-trees are connected by lines of the same color, while branches leading to distinct sub-trees are marked by a dashed line.

**Figure S5.** **Principal component analysis (PCA) in 3 dimensions of *Mycobacterium bovis* lineages according to the reduced dataset (*n*=1,201).** The PCA graphs were constructed using the SNP (single nucleotide polymorphism) matrix of the 1,201 *Mycobacterium bovis* genomes. (**A**) PCA colored according to the proposed *M. bovis* lineages (Lb1, Lb2, Lb3 and Lb4) and unknown clusters (unknown 1, 2 and 3). (**B**) PCA colored according to the proposed lineages Lb1, Lb2 and Lb3, unknown clusters (unknown 1, 2 and 3) and clusters of *M. bovis* genomes emerging from nodes G, H and I of Lb4 as depicted in the phylogenetic tree in Figure 2. Component 1= 52.0%; Component 2 = 22.1%, Component 3 = 9.8%.

**Figure S6.** **Principal component analysis (PCA) of the original/complete *Mycobacterium bovis* genomes dataset (*n*=1,969).** (**A**) PCA colored according to the proposed lineages (Lb1, Lb2, Lb3 and Lb4) and unknown clusters (unknown 1, 2 and 3). (**B**) PCA colored according to the proposed lineages Lb1, Lb2 and Lb3, unknown clusters (unknown 1, 2 and 3), and clusters of *M. bovis* genomes emerging from what would be the equivalent to nodes G, H and I of Lb4, depicted in the phylogenetic tree of Figure 2 (reduced dataset of 1,201 *M. bovis* genomes), in a phylogenetic tree generated with the original/complete dataset.

**Figure S7.** **Principal component analysis (PCA) in 3 dimensions of *Mycobacterium bovis* lineages according to the original/complete dataset (*n*=1,969).** The PCA graphs were constructed using the SNP (single nucleotide polymorphism) matrix of the 1,201 *Mycobacterium bovis* genomes. (**A**) PCA colored according to the proposed *M. bovis* lineages (Lb1, Lb2, Lb3 and Lb4) and unknown clusters (unknown 1, 2 and 3). (**B**) PCA colored according to the proposed lineages Lb1, Lb2 and Lb3, unknown clusters (unknown 1, 2 and 3), and clusters of *M. bovis* genomes emerging from what would be the equivalent to nodes G, H and I of Lb4, depicted in the phylogenetic tree of Figure 2 (reduced dataset of 1,201 *M. bovis* genomes), in a phylogenetic tree generated with the original/complete dataset. Component 1= 52.0%; Component 2 = 22.1%, Component 3 = 9.8%.

**Figure S8.** **Cluster of orthologous groups (COGs) of predicted proteins carrying SNP (single nucleotide polymorphisms) markers of lineages and unknown clusters of *Mycobacterium bovis*.** Lb1: Lineage 1 of *M. bovis*; Lb2: Lineage 2 of *M. bovis*, Lb3: Lineage 3 of *M. bovis*. Lb4 (lineage 4 of *M. bovis*) does not carry any unique SNP marker. **D:** Cell cycle control, cell division, chromosome partitioning; **M:** Cell wall/membrane/envelope biogenesis; **N:** Cell motility; **O:** Post-translational modification, protein turnover, and chaperones; **T:** Signal transduction mechanisms; **U:** Intracellular trafficking, secretion, and vesicular transport; **V**: Defense mechanisms; **W**: Extracellular structures; **Y:** Nuclear structure; **Z:** Cytoskeleton; **A:** RNA processing and modification; **B:** Chromatin structure and dynamics; **J:** Translation, ribosomal structure and biogenesis; **K:**Transcription; **L:** Replication, recombination and repair; **C:** Energy production and conversion; **E:** Amino acid transport and metabolism; **F:** Nucleotide transport and metabolism; **G:** Carbohydrate transport and metabolism; **H:** Coenzyme transport and metabolism; **I:** Lipid transport and metabolism; **P:** Inorganic ion transport and metabolism; **Q:** Secondary metabolites biosynthesis, transport, and catabolism; **R:** General function prediction only; **S:** Function unknown.


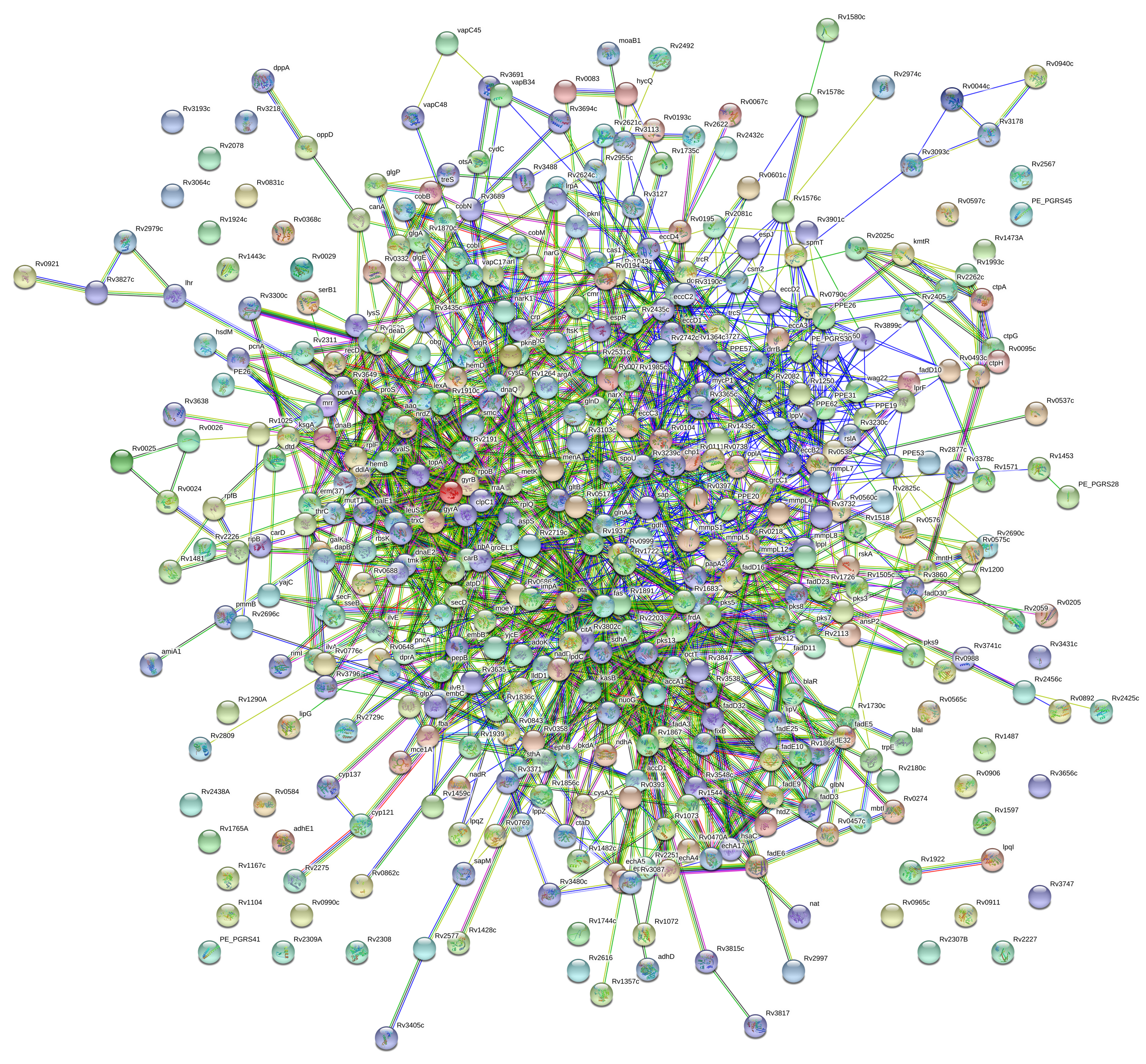


**Figure S9. Protein-protein interaction network of genes carrying SNP markers of cluster unknown 1 of *Mycobacterium bovis.*** STRING protein network from the corresponding proteins of genes carrying unique SNPs (single nucleotide polymorphisms) of the Cluster "unknown 1" (Malawi genomes) of *M. bovis* (Table S3). As Cluster “unknown 1” is composed of only 3 *M. bovis* genomes, the number of unique SNPs is very high, precluding detailed analyses.


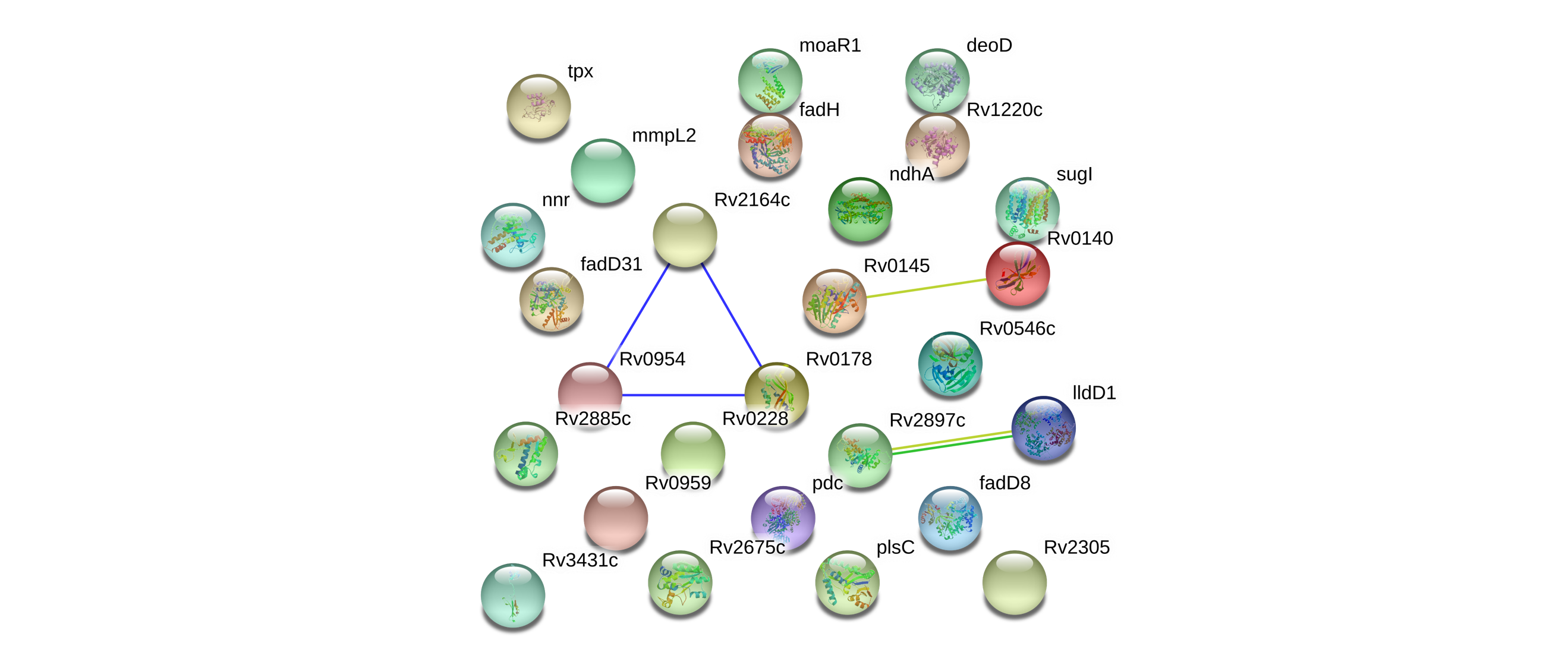


**Figure S10. Protein-protein interaction network of genes carrying SNP markers of cluster unknown 2 of *Mycobacterium bovis.*** STRING protein network from the corresponding proteins of genes carrying unique SNPs (single nucleotide polymorphisms) of the Cluster "unknown 2" (Russia and Germany genomes) of *M. bovis* (Table S3).


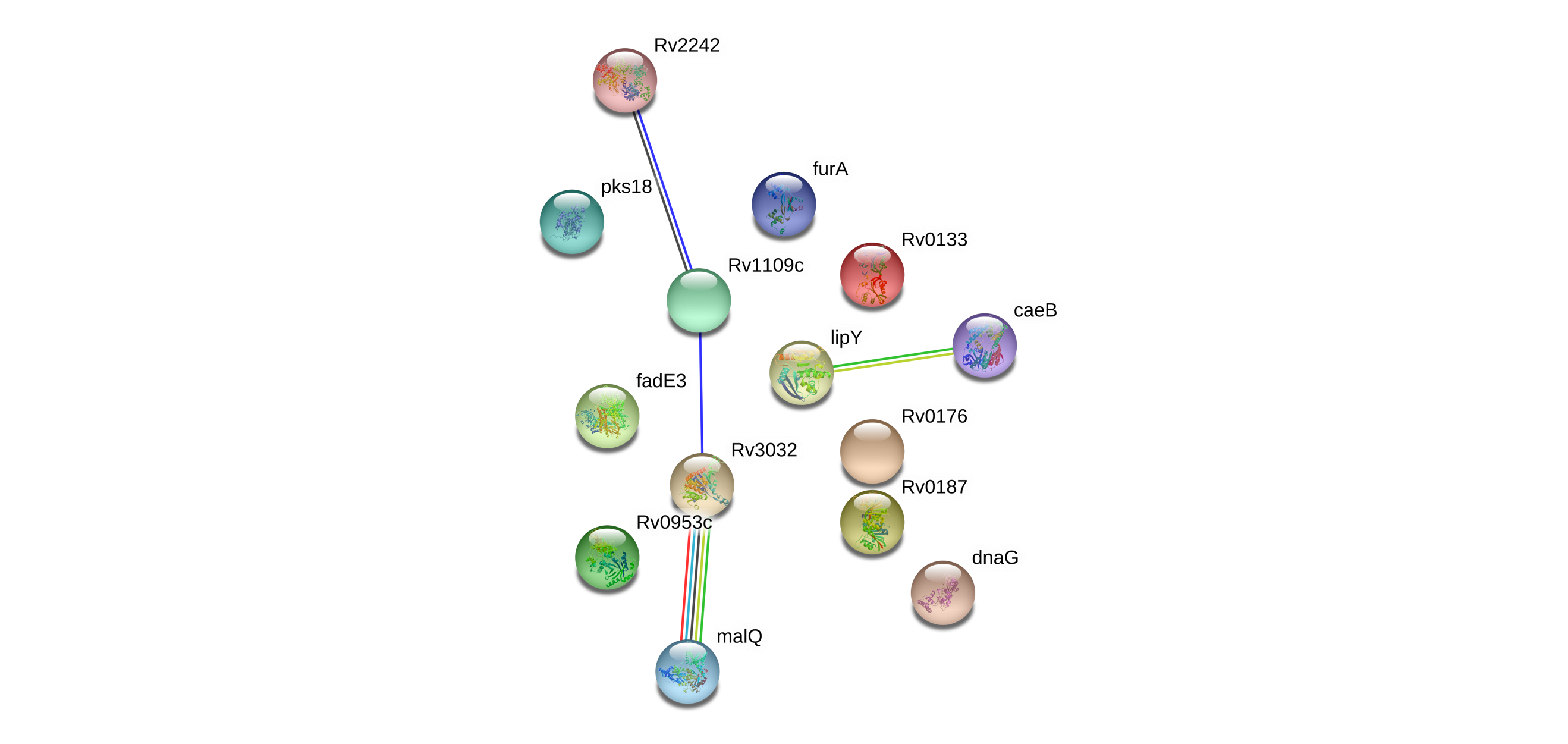


**Figure S11. Protein-protein interaction network of genes carrying SNP markers of Lb1 of *Mycobacterium bovis.*** STRING protein network from the corresponding proteins of genes carrying unique SNPs (single nucleotide polymorphisms) of the Lineage 1 of *M. bovis* (Lb1) (Table S3).


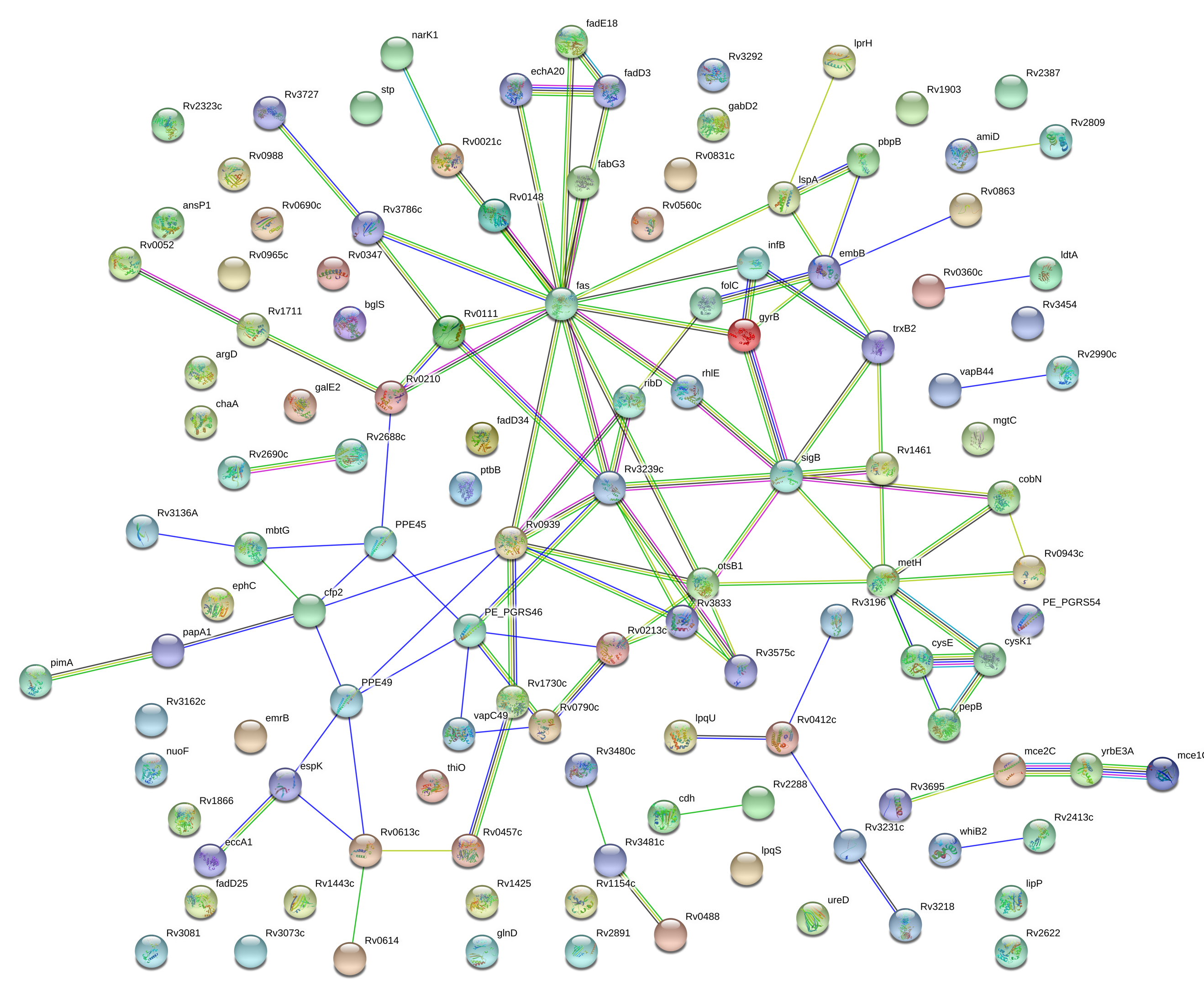


**Figure S12. Protein-protein interaction network of genes carrying SNP markers of Lb2 of *Mycobacterium bovis.*** STRING protein network from the corresponding proteins of genes carrying unique SNPs (single nucleotide polymorphisms) of the Lineage 2 of *M. bovis* (Lb2) (Table S3).


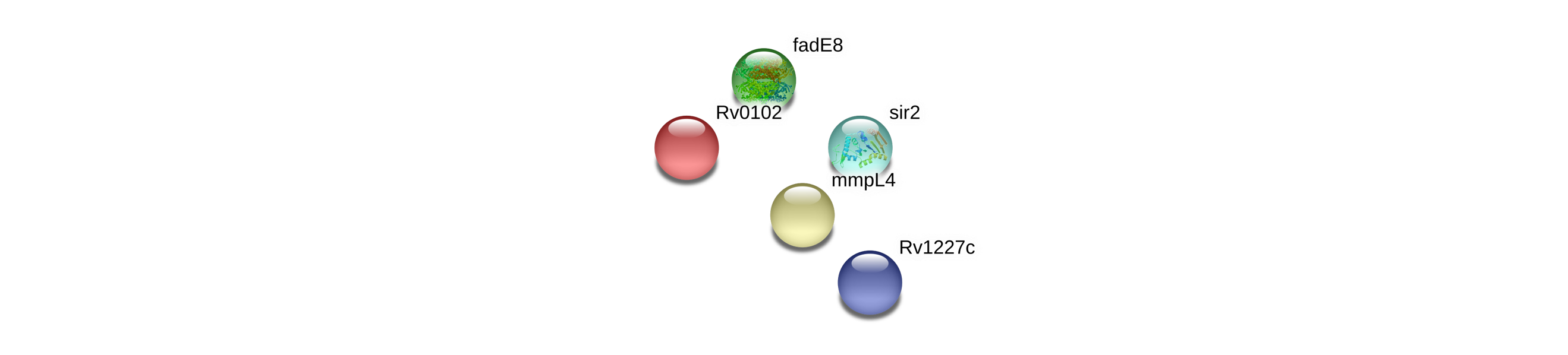


**Figure S13. Protein-protein interaction network of genes carrying SNP markers of Lb3 of *Mycobacterium bovis.*** STRING protein network from the corresponding proteins of genes carrying unique SNPs (single nucleotide polymorphisms) of the Lineage 3 of *M. bovis* (Lb3) (Table S3).
